# Role of health literacy on lifestyle and glycemic control among women with diabetes during pregnancy: a cross-sectional study

**DOI:** 10.3389/fpubh.2024.1418525

**Published:** 2024-10-09

**Authors:** Towhid Babazadeh, Sara Pourrazavi, Zahra Ardeshiri, Akbar Nadi, Khalil Maleki Chollou

**Affiliations:** ^1^Department of Public Health, Sarab Faculty of Medical Sciences, Sarab, Iran; ^2^Research Center of Psychiatry and Behavioral Sciences, Tabriz University of Medical Sciences, Tabriz, Iran; ^3^Department of Health Education & Promotion, Tabriz University of Medical Sciences, Tabriz, Iran; ^4^Department of Nursing, Sarab Faculty of Medical Sciences, Sarab, Iran

**Keywords:** health literacy, lifestyle, pregnant women, diabetes during pregnancy, Iran

## Abstract

**Background:**

The present study aimed to explore the impact of health literacy on the lifestyle of women with diabetes during pregnancy.

**Methods:**

A cross-sectional study assessed the influence of some demographic characteristics and health literacy dimensions in predicting lifestyle and glycemic control in a sample of 230 women with diabetes during pregnancy. The data collection included a demographic form, a health literacy scale, and a lifestyle questionnaire. The data were analyzed using a one-way ANOVA and Pearson’s correlation coefficient. The predictors were determined using a hierarchical linear regression analysis.

**Results:**

The participants had an average age of 27.74 years (SD = 6.54) and an average HbA1c level of 6.93% (SD = 1.93). Approximately 51.0% of the variation in lifestyle can be explained by health literacy (HL) and demographic variables (*p*-value <0.05). In addition, approximately 15.0% of the variation in HbA1c can be explained by health literacy, lifestyle, and demographic variables (*p*-value <0.05).

**Findings:**

According to our findings, decision-making was found to be the strongest predictor of lifestyle. This study provides valuable information for nurses and other healthcare providers to help empower pregnant women to increase their health literacy and improve their lifestyle.

## Introduction

Pregnancy is a significant time in a woman’s life, and taking care of her health is crucial for the wellbeing of both mother and child (1). During this period, mothers face various potential dangers, and one of these risks is diabetes during pregnancy (2). ‘Diabetes in pregnancy’ is defined as any degree of glucose intolerance with onset or first recognition during pregnancy (3). The studies showed that diabetes during pregnancy is associated with adverse short- and long-term risks for women and their offspring (4), and a meta-analysis showed that diabetes during pregnancy is associated with cardiovascular disease (5). The conducted study among pregnant American women reported that among individuals with a singleton first live birth from 2011 to 2019, the rates of diabetes during pregnancy increased (6). A study conducted in the Eastern Mediterranean region shows a high prevalence of diabetes during pregnancy. According to this report, the prevalence rates for Pakistan, Qatar, Bahrain, and Iran are 15.3, 14.7, 12.2, and 8.6%, respectively (7).

The biological changes that occur during pregnancy can have a significant impact on women’s lifestyle and overall health (8). A healthy lifestyle is a multi-dimensional pattern of self-initiated feelings and behaviors aiming at ensuring an individual’s health, self-actualization, and self-accomplishment (9–11). Pregnant women’s lifestyle encompasses various aspects, such as their work–life balance, resting patterns, nutritional choices, stress management techniques, communication styles, and prenatal care (11). Having a health-promoting lifestyle during pregnancy can be effective in maintaining women’s health in order to deliver healthy infants and decrease the risk of low birth weight (12).

One strategy to enhance a healthy lifestyle is to boost information and encourage health literacy (HL) among individuals. According to the World Health Organization (WHO) definition, HL is “the ability to access, understand, evaluate, and communicate information as a way to promote, maintain, and improve health in a variety of settings across the life course” (13, 14). Increasing awareness and preparedness during pregnancy can contribute to a smoother journey for the mother with fewer complications. It presents an excellent opportunity to educate and counsel pregnant women, making them aware of the benefits of embracing a healthy lifestyle (15).

HL helps pregnant women acquire the necessary information about diabetes during pregnancy, take an active role in managing their condition, and make better decisions for their own and their baby’s health. In general, HL plays a crucial role in managing diabetes during pregnancy and improving maternal and fetal outcomes (16, 17). Therefore, this study aimed to explore the impact of HL on the lifestyle choices of women with diabetes during pregnancy. By understanding the importance of HL, healthcare professionals, particularly midwives, can play a significant role in providing counseling services to enhance HL and promote healthier lifestyle choices among pregnant women with diabetes during pregnancy (16).

## Methodology

The current cross-sectional study was conducted in Sarab County, a mountainous county in East Azerbaijan, the northwestern part of Iran. The study population comprised pregnant women receiving care at governmental primary health care service centers (HCSCs) in urban areas of the county. The District Health System (DHS) in Iran is responsible for providing primary health care (PHC) services to specific populations. The main objective of the DHS is to promote the wellbeing of the community. In accordance with Iran’s National Health Care System (NHCS), healthcare centers are categorized as urban and rural healthcare centers. These healthcare centers are tasked with delivering various primary healthcare services, including health education, maternal and child health services, health nutrition education, and, more, to the populations they serve.

A multistage cluster sampling technique was utilized to recruit a total of 230 participants from HCSCs. Initially, four HCCs were chosen as clusters in the first stage of sampling. Subsequently, respondents were randomly selected from these four HCCs in the second stage.

The inclusion criteria for the study were as follows: pregnant women aged 18 years and older, at least 24 weeks into their pregnancy, diagnosed with gestational diabetes according to their health records, treated for diabetes during pregnancy either through diet or medications, and participants from all literacy levels. Exclusion criteria included individuals with a psychotic disorder, people with a history of type 1 and type 2 pregestational diabetes, and those unwilling to participate in the study.

The sample size was determined by considering information from a similar study (18) and a confidence level of 99% with Z = 2.57. The parameters used for calculations were SD = 3.79 and mean = 9.95, based on 223 samples. However, to account for potential sample dropouts, the final sample size was adjusted to be larger than the originally calculated amount. Data were collected from participants through interviews carried out by trained research assistants. The interviews for the study were conducted in the consulting room of the health centers over a period of 6 months. Each interview had a duration of 20–25 min. Prior to administering the questionnaires, the study objectives were explained to the patients, and they were provided with an informed consent form to complete. This ensured that the patients understood the purpose of the study and voluntarily agreed to participate.

## Instrument

### Demographic characteristics

The following demographic data were obtained: age, education, husband’s education, job, husband’s job, and history of high-risk pregnancy.

### HL scale

The scale is a valid and reliable instrument that was developed by Montazeri et al. to assess HL among Iranian adults (19). The HL instrument is composed of five subscales as follows:

#### Reading health information

The dimension of reading health information was measured using a four-item subscale (*α* = 0.72) on a five-point Likert-type scale ranging from 1 to 5 (1 = completely difficult through 5 = completely easy). An example of this domain was as follows: “reading health education materials (booklet, pamphlet, and educational brochures) was easy for me.” The total scores possible ranged from 5 to 20, with higher scores indicating stronger reading abilities.

#### Understanding health information

To measure the understanding of health information, a total of seven items were used, such as “I can acquire the required health and medical information from different sources.” Participants rated each item on a 5-point scale ranging from 1 to 5, with 1 indicating complete difficulty and 5 indicating complete ease. The subscale demonstrated good internal consistency with Cronbach’s alpha coefficient of 0.86. The theoretical range for this subscale was 5 to 35, with higher scores indicating a greater ability to understand health information.

#### Appraisal of health information

The appraisal of health information was assessed using four items, such as “I can get information about healthy nutrition.” Participants rated each item on a five-point Likert-type scale, ranging from 1 (never) to 5 (always). The internal consistency of this measure was good, with Cronbach’s alpha coefficient of 0.77. The total score on the appraisal of health information index could range from 5 to 20, with a higher score indicating a higher level of appraisal of health information.

#### Ability to access health information

The subscale for assessing the ability to access health information consisted of six items, such as “I can obtain information about my illness.” Participants used a five-point Likert-type scale to rate each item, with the following scale: always = 5, most of the time = 4, sometimes = 3, seldom = 2, and never = 1. The total possible scores on this subscale ranged from 5 to 30, with a higher score indicating a greater ability to access health information. The internal consistency of this subscale was high, with Cronbach’s alpha coefficient of 0.86.

#### Decision-making

The decision-making subscale consisted of 12 items and had a high internal consistency, with Cronbach’s alpha coefficient of 0.89. This subscale was developed to measure the ability to make decisions regarding health-related behaviors. Examples of items included the following: “I avoid doing things or taking materials that might increase my weight” and “I do not stop taking medications without my doctor’s permission, even if the symptoms of the disease would disappear.” Participants rated each item on a five-point Likert-type scale, with the following scale: always = 5, most of the time = 4, sometimes = 3, seldom = 2, and never = 1. Higher scores on this subscale indicated a greater ability in decision-making regarding health-related behaviors.

### Lifestyle scale

Finally, we utilized a valid and reliable scale to measure lifestyle developed by Mohsen et al. (20). The instrument comprised four dimensions: psychological health (7 items; *α* = 0.88), weight control and nutrition (7 items; α = 0.85), physical activity (7 items; α = 0.87), and avoidance from drugs (6 items; α = 0.79). For all four dimensions, participants used a four-point Likert-type scale to rate the items, ranging from 1 (never) to 4 (always).

### Glycemic control

The outcome variable in the study was the HbA1c value of patients, which is a measure of glycemic control. The patient’s medical records were used to record the most recent HbA1c value. According to the International Expert Committee, an HbA1c ≥6.5% was used as a measure for glycemic control (21, 22).

## Data analysis

We performed all the analyses using SPSS 16 (SPSS Inc., Chicago, IL, United States) and presented the data by mean (SD) and frequency (percent) for quantitative and qualitative variables, respectively. Before analyzing the data, the Kolmogorov–Smirnov test was used to determine the normality of the data. The data distribution was normal according to the significance level of more than 0.05 of the Kolmogorov–Smirnov test. Therefore, the quantitative variables were analyzed using bivariate analyses (i.e., the one-way ANOVA test). Pearson’s correlation coefficient was used to measure the relationship between lifestyle and HL dimensions. We also used the Kolmogorov–Smirnov test to test the normality.

A two-step hierarchical linear regression analysis was conducted to determine which variables can predict lifestyle. In Step 1, the covariates (i.e., age, education, husband’s education, job, husband’s job, and history of high-risk pregnancy) were entered into the models. HL dimensions were involved in the second step, along with the demographic variables. A *p-value of* < 0.05 was considered significant.

## Results

A total of 230 individuals willingly volunteered to take part in the study. Table 1 provides an overview of the participants’ demographic characteristics, as well as their associations with lifestyle and HL. The average age and HbA1c of the participants were 27.74 years (standard deviation = 6.54) and 6.93% (standard deviation = 1.93), respectively, with a majority falling into the under-30 age group. Only 14.3% of the participants were employed, and 17.8% had a history of high-risk pregnancy. As displayed in Table 1, a statistically significant association was found between lifestyle and participants’ education (*p*-value <0.001), husband’s education (*p*-value <0.001), job (*p*-value = 0.032), and husband’s job (*p*-value = 0.020). The findings indicated a statistically significant association between HL and the education levels of pregnant women (*p*-value <0.001), their husband’s education levels (*p*-value <0.001), their jobs (*p*-value <0.001), and their husband’s jobs (*p*-value <0.001).

**Table 1:** Demographic characteristics of the study participant and their association with lifestyle and health literacy.

Variables		F (%)	Lifestyle	*p*-value *	Health literacy	*p*-value
			Mean ± standard deviation		Mean ± standard deviation	
Age groups (years) *	30 >	149 (64.8)	79.74 ± 8.84	0.152	76.51 ± 22.50	0.191
	30 ≤	81 (35.2)	77.55 ± 12.01		81.22 ± 27.07	
Education *	Under diploma	99 (43.0)	75.05 ± 10.21	< 0.001	68.11 ± 18.61	< 0.001
	Diploma and higher	131 (57.0)	81.96 ± 8.97		91.48 ± 24.99	
Husband’s education *	Under diploma	91 (39.6)	75.54 ± 11.15	< 0.001	71.46 ± 20.33	< 0.001
	Diploma and higher	139 (60.4)	81.21 ± 8.68		88.42 ± 26.70	
Job *	Housewife	197 (85.7)	82.45 ± 14.23	0.032	63.81 ± 23.57	< 0.001
	Employed	33 (14.3)	78.39 ± 9.15		80.57 ± 23.83	
Husband’s job *	Self-employment	170 (73.9)	78.05 ± 9.47	0.020	67.36 ± 22.72	< 0.001
	Employed	60 (26.1)	81.58 ± 11.39		81.98 ± 23.97	
History of high-risk pregnancy *	Yes	41 (17.8)	78.07 ± 10.94	0.530	84.87 ± 26.01	0.053
	No	189 (82.2)	79.16 ± 9.93		76.71 ± 23.93	

*Independent T-test.

In Table 2, the bivariate correlations between HL dimensions and lifestyle are displayed. Pearson’s correlation coefficient was used to examine these relationships. The results indicate that lifestyle demonstrated statistically significant correlations with all HL dimensions (*p*-value <0.05).

**Table 2:** Bivariate correlation between health literacy dimensions and lifestyle.

Variables	1	2	3	4	5	6	Mean ± standard deviation
1 = Reading health information	1						10.01 ± 4.31
2 = Ability to access health information	0.792*	1					14.23 ± 4.68
3 = Understanding health information	0.822*	0.724*	1				15.46 ± 6.41
4 = Appraisal of health information	0.610 *	0.656*	0.719*	1			9.85 ± 3.47
5 = Decision-making	0.620*	0.558*	0.754*	0.556*	1		28.60 ± 9.13
6 = Lifestyle	0.510*	0.471*	0.568*	0.494*	0.681*	1	78.97 ± 10.10

^*^Correlation is significant at the 0.01 level (two-tailed).

The effects of demographic variables and HL dimensions on lifestyle were assessed using a hierarchical linear regression analysis (Table 3). In step 1, demographic variables explained significantly 14.6% of the variation in lifestyle (*p*-value <0.05). The education of participants (ß = 0.244; *p*-value = 0.012) was identified as a demographic characteristic that can predict lifestyle. An additional 36.14% of the variation in lifestyle was explained by HL dimensions as predictor variables (step 2) (*p*-value >0.05). Overall, when considering both demographic characteristics and HL dimensions, the analysis found that they collectively accounted for 51.0% of the variation in lifestyle.

**Table 3:** Hierarchical linear regression for prediction lifestyle through demographic characteristics and health literacy dimensions.

Variables	ß	R2 change	F change	Standard error	*p*-value
Step 1					
Age groups (years)	−0.124	0.146	6.33	1.41	< 0.001
Education	0.244*			1.50	
Husband’s education	0.139			1.55	
Job	−0.076			1.95	
Husband’s job	−0.023			1.59	
History of high-risk pregnancy	−0.056			1.78	
Step 2					
Age groups (years)	−0.093	0.364	32.39	1.14	< 0.001
Education	0.040			1.24	
Husband’s education	0.122 *			1.22	
Job	−0.048			1.60	
Husband’s job	0.002			1.24	
History of high-risk pregnancy	−0.073			1.37	
Reading health information	0.041			0.23	
Ability to access health information	0.039			0.18	
Understanding health information	0.04			0.17	
Appraisal of health information	0.158*			0.208	
Decision-making	0.587*			0.081	
Total R2	–	0.510	–	–	–
Adjusted R2	–	0.485	–	–	–

*p < 0.05.

We used hierarchical linear regression to predict HbA1C. According to Table 4, in step 1, demographic variables were significant predictors of HbA1C (*p*-value = 0.017). The jobs of participants (ß = 0.181; *p*-value = 0.012) were a predictor of HbA1C. In step 3, HL dimensions and lifestyle were added, which explained an additional 12.8% of the variance (*F* = 5.720; *p*-value <0.001). In total, demographic characteristics, HL dimensions, and lifestyle were able to explain 19.4% of the variance in HbA1C.

**Table 4:** Hierarchical linear regression for prediction HbA1c through demographic characteristics, health literacy dimensions, and lifestyle.

Variables	ß	R2 change	F change	Standard error	*p*-value
Step 1					
Age groups (years)	0.118	0.066	2.63	0.159	0.017
Education	0.083			0.169	
Husband’s education	0.088			0.175	
Job	0.181*			0.220	
Husband’s job	0.104			0.159	
History of high-risk pregnancy	0.049			0.201	
Step 2					
Age groups (years)	0.063	12.8	5.720	0.155	< 0.001
Education	0.028			0.173	
Husband’s education	0.146			0.170	
Job	0.134			0.223	
Husband’s job	0.090			0.172	
History of high-risk pregnancy	0.029			0.191	
Reading health information	0.050			0.033	
Ability to access health information	0.060			0.025	
Understanding health information	0.092			0.024	
Appraisal of health information	0.005			0.029	
Decision-making	0.087			0.013	
Lifestyle	0.331			0.009	
Total R2	–	19.4	–	–	–
Adjusted R2	–	14.9	–	–	–

*p < 0.05.

## Discussion

Blood sugar control in women with diabetes during pregnancy is regarded as an established challenge. This can be more challenging when diabetic pregnant women have limited HL. The results of this study reinforce the importance of improving healthy lifestyle habits by enhancing HL in pregnant women with diabetes.

Our findings revealed a significant relationship between HL and the lifestyles of pregnant women, as well as their husband’s education levels and occupations. The current study reported that education level is associated with possessing a higher level of HL and a healthy lifestyle. Similarly, studies have shown a significant relationship between the level of education and the level of HL and lifestyle (14, 23). It has been reported that the level of education is one of the important predictors of HL (14, 23, 24). Education can lead to increased consciousness and behaviors related to healthy lifestyles, and this in turn may contribute to improving HL (23). On the other hand, working women may study more and receive a wider education due to their jobs, which can provide them with knowledge about health, prevention, and self-care. Therefore, considering the important role of education in HL and improving lifestyle, including educational programs related to healthy lifestyles, especially since childhood, can play an important role in promoting the health of pregnant women.

The findings emphasized the importance of HL as a predictor of healthy lifestyles among women with diabetes during pregnancy. Our results showed that of the HL dimensions, the appraisal of health information and decision-making were significant predictors of lifestyle and explained approximately 36.14% of the variance in the model.

According to the literature, low HL can be associated with a wide range of negative health outcomes (25–27). They have found that HL can lead to a healthy lifestyle and improve the quality of life (24, 26). Women with diabetes during pregnancy are in a critical phase of their lives and have unique information needs compared to other pregnant women (16). HL among pregnant women is considered an important factor that can affect pregnancy complications and the health of both mothers and their children (24). The ability to acquire, understand, evaluate, and apply health information enables them to make correct judgments and decisions regarding healthcare, disease prevention, and health promotion in their daily lives, ultimately helping them go through their pregnancy with fewer complications (25).

Studies have shown that low levels of HL are associated with insufficient self-management skills and an increased likelihood of adverse health outcomes, such as not taking daily vitamins during pregnancy and experiencing uncontrolled diabetes (25, 27, 28). In contrast, pregnant women with high HL are more likely to seek consultations, undergo regular health checks, take folic acid, and be more physically active (29). Therefore, increasing the level of HL plays a role in changing the lifestyles of pregnant women, and improving their health and pregnancy can be a good opportunity to educate and advise pregnant women and make them aware of the benefits of a healthy lifestyle (16).

Our findings indicated the importance of HL and lifestyle in blood sugar control of women with diabetes during pregnancy. It has been proven that inadequate blood sugar management in diabetic patients increases the likelihood of health complications and the likelihood of severe illness and death (30). The main indicator of blood sugar control is HbA1c (31). According to studies, lower HbA1c levels are associated with fewer health complications and lower mortality among people with diabetes (31, 32). Studies have shown that self-care behaviors and HL are associated with blood sugar control (31, 33). In fact, one of the main determinants of HbA1c control is self-care behaviors (33). HL plays an important role in the adaptation of diabetic patients to self-care behaviors and diabetes outcomes (31). HL enables diabetic individuals to access the necessary information to control their blood sugar and manage their health (34).

## Conclusion

According to our findings, education level is associated with possessing a higher level of HL and a healthy lifestyle. In addition, the appraisal of health information and decision-making as dimensions of HL are significant predictors of lifestyle change. Our study has revealed that HL dimensions and lifestyle were able to explain sugar control among diabetic pregnant women. Therefore, our findings support and extend previous reports on the lifestyle and HL in health outcomes of pregnant women with diabetes and offer additional information on the importance of improving healthy lifestyle habits by enhancing HL among them. The results of this study provide important insights for health professionals and health policymakers and highlight the importance of developing strategies to improve the HL of pregnant women.

### Limitations

First, while the present study indicates a causal relationship between variables, it is important to note that the cross-sectional design of the data prohibits making causal inferences. Instead, it can only establish correlations that exist at a specific moment between the observed constructs. Second, the measurements in this study were self-reported, which is a commonly used method for measuring psychosocial variables in primary care research. However, it is important to acknowledge that self-reporting may not be perfect as it relies on individuals’ subjective accounts. Nonetheless, self-report measures provide valuable insights into participants’ experiences, thoughts, and behaviors in primary care research.

## Data Availability

The raw data supporting the conclusions of this article will be made available by the authors, without undue reservation.
